# Characterization of the active response of a guinea pig carotid artery

**DOI:** 10.3389/fbioe.2022.924019

**Published:** 2022-08-26

**Authors:** Álvaro Navarrete, Pablo Varela, Miguel López, Claudio M. García-Herrera, Diego J. Celentano, Bernardo Krause

**Affiliations:** ^1^ Departamento de Ingeniería Mecánica, Universidad de Santiago de Chile, Santiago, Chile; ^2^ Departamento de Ingeniería Mecánica y Metalúrgica, Pontificia Universidad Católica de Chile, Santiago, Chile; ^3^ Instituto de Ciencias de la Salud, Universidad de O’Higgins, Rancagua, Chile

**Keywords:** smooth muscle cells, active response, mechanochemical model, isometric contraction, finite element method

## Abstract

This work presents a characterization of the active response of the carotid artery of guinea pig fetuses through a methodology that encompasses experiments, modeling and numerical simulation. To this end, the isometric contraction test is carried out in ring samples subjected to different levels of KCl concentrations and pre-stretching. Then, a coupled mechanochemical model, aimed at describing the smooth cell behavior and its influence on the passive and active mechanical response of the vascular tissue, is calibrated from the experimental measurements. Due to the complex stress and strain fields developed in the artery, a finite element numerical simulation of the test is performed to fit the model parameters, where those related to the phosphorylation and dephosphorylation activity along with the load-bearing capacity of the myosin cross-bridges are found to be the most predominant when sensitizing the active response. The main strengths of the model are associated with the prediction of the stationary state of the active mechanical response of the tissue through a realistic description of the mechanochemical process carried out at its cellular level.

## 1 Introduction

Smooth muscle cells (SMC) consist of spindle-shaped, elongated, and uninucleated cells embedded in an extracellular matrix (ECM) of fibrous nature (Kao and Carsten, 1997). SMCis distributed in sheets or layers in hollow and tubular organs such as the stomach, intestines, airways, and blood vessels, among others (Clinton, 2003). These layers work as a whole with each other due to the presence of intercellular connections (gap junctions) between their cell membranes (Figueroa and Duling, 2009), playing a fundamental role in highly specialized physiological processes related to contraction/relaxation cycles and providing mechanisms to regulate both fluid movements along with structural characteristics such as its shape and size (Frismantiene et al., 2018).

Focusing on vascular smooth muscle cells, they are the main component of medium and large-sized arteries and are in the central region of the artery wall (tunica media) (Hu and Zhang, 2019). Among their physiological functions are vasoconstriction, vasodilation, and both control and maintenance of the ECM (Bacakova et al., 2018). Therefore, this type of cell contributes to maintaining structural coherence and controlling the diameter of blood vessels in the presence of physiological stimuli (Metz et al., 2012). Dynamic changes in vascular diameter (contraction/relaxation) are primarily associated with intracellular free calcium concentration [Ca^+2^] (Touyz et al., 2018). In muscular vessels, SMC and endothelial cells are responsible for vasomotion when they are subjected to mechanical stress, using intraluminal pressure variation and shear stress, whose effects trigger variation in calcium concentration (Koenigsberger et al., 2006).

In addition to the features above, vascular SMC dysfunction is actively involved in the pathogenesis of several diseases. One of the most studied is hypertension, which corresponds to a systemic and chronic condition manifested by high arterial pressure and increased vascular resistance (Frismantiene et al., 2018). In addition to the endothelial dysfunction and arterial remodeling, which includes changes in the vascular SMC phenotype (Touyz et al., 2018), hypertension is characterized by an abnormal contraction/relaxation process, where an increased contraction level and a decreased relaxation are observed (Szasz and Tostes, 2016). This behavior is derived from a disorder in the vascular SMC response to the increasing vasoconstrictor signaling (Frismantiene et al., 2018). On the other hand, intrauterine growth restriction (IUGR), which corresponds to an affection in which the fetal growth is below the potential size given by genetic aspects, can occur due to multiple factors, such as maternal, paternal, fetal, genetic, or a combination of them (Sharma et al., 2016). This intrauterine noxa induces long-lasting changes in the vascular SMC reactivity, which is due to a reduced maximum [Ca^+2^] activated force, triggered likely by a decrease in contractile proteins activated in an excitation/contraction cycle (Christie et al., 2018).

Mechanochemical stimuli, in general, determine SMC contraction and are mainly mediated by an increase in the calcium-free intracellular concentrations [Ca^+2^]. The latter are regulated by calcium channels in the cell membrane that allow the flux between the extracellular and intracellular milieu and channels located within the sarcoplasmic reticulum (SR) membrane (Murtada et al., 2010). Once an increase in the intracellular calcium concentration is generated, it binds to a protein called calmodulin (CaM), forming a calcium-calmodulin complex (Ca-CaM) that binds to and activates the myosin light-chain kinase (MLCK). In response to the activation of this enzyme, the myosin light chains (MLC) are phosphorylated, leading to an attachment of the cross-bridges of the actin filament and, consequently, to the SMC contraction. On the other hand, SMC relaxation occurs when the concentration of [Ca^+2^] decreases below a critical level while being pumped out of the cell or into the SR. The [Ca^+2^] is then released from the calmodulin (CaM), and the myosin light-chain phosphatase (MLCP) removes phosphate from the MLC, thus detaching the cross-bridges from the actin filament and relaxing the smooth muscle (Guyton and Hall, 2011).

Several tests focused on determining the history-effect have been performed to describe the main aspects that trigger the active contractile response of SMC. A typical *in-vitro* experiment used to obtain active stress-stretch is the isometric contraction test (Murtada et al., 2016; Murtada et al., 2017; Caulk et al., 2019), where a sample is pre-stretched at different levels of deformation, measuring the active force evolution from the instant when it is stimulated to contract, and keeping fixed its length (Murtada et al., 2012). An arterial ring section is typically used for this test, which allows the study of arteries of small sizes. Conversely, the quick-released experiment can determine the force-velocity relationship, in which once the muscle strip reaches a specific isometric force, it is released from one of its ends until a particular constant afterload is achieved. Depending on the afterload value, the muscle tissue stretches or contracts, and the velocity with which it does it is accounted for along with the afterload force (Chitano et al., 2017). In addition, by imposing length-controlled quick stretches at different times, the active stress and its evolution can be determined (Dillon et al., 1981). (Tomalka et al., 2017) investigated the biomechanical SMC properties in porcine stomaches using the previously described test, determining the force-length and force-velocity relations under active muscle shortening and stretching to study the conditions associated with the long dimensional changes underwent by the stomach and their SMC. Moreover, changes in the mechanical properties can be related to an alteration of the normal SMC function. (Fujishige et al., 2002). studied the influence on the mechanical properties of the calponin protein, an SMC-specific actin-binding protein involved in regulating the SMC contraction. To this end, the aortic tissue in mice with a mutated essential calponin locus was analyzed. Using the mechanical test mentioned above, the force development under different stimulation degrees associated with specific KCl concentrations has been reported, suggesting that calponin plays a fundamental role in controlling the SMC contraction. (Kim et al., 2015). described the passive and active mechanical behavior of the porcine small intestine, performing tensile tests to obtain the passive response, along with isometric and isotonic experiments to capture the active response, determining maximum loads and stresses in the tensile test and isometric experiment and maximum velocity muscular contraction from the isotonic experiment. (Koenigsberger et al., 2008), through a theoretical model, establish the effect of different experimental conditions (isometric, isobaric, isotonic, or cyclic pressure variations) on calcium concentration and arterial radius dynamics, concluding that the most realistic scenario (with respect to the *in vivo* situation, referred to as cyclic pressure condition) is given by the isobaric state, where similar levels of calcium concentration were observed concerning a control case.

Multiple efforts have been made to describe through models the active response of biological soft tissue. In work performed by (Rachev and Hayashi, 1999), the effect of active response on residual deformations was studied. For this purpose, a phenomenological model was proposed, considering two factors: one depending on the stimulus applied to contractile activity and another that depends on the relationship between the deformation and the active stress determined via the isometric contraction test. On the other hand, theoretical approaches that consider SMC mechanisms have been used to predict the tissue’s contraction and relaxation observed to a macrostructural level. Most of the research works have been focused on the proposal of models that consider, according to the relative sliding filament theory, that the active force is generated by cross-bridges interaction from the myosin to the actin filaments (Huxley and Niedergerke, 1958). Another remarkable aspect of these models is the use of averaged characteristic cross-bridges attached to the actin filaments instead of the sum of individual contributions. About purely mechanical approaches, (Gestrelius and Borgstrom, 1986), developed a model in which the transfer of energy through the cross-bridges can be considered like a “friction-clutch” mechanism. The results obtained with this formulation agree with experimental observations under isometric and isotonic conditions, particularly those of the quick-release experiment when the afterload is greater than the isometric force, for which previous models do not describe the experimental findings satisfactorily. Several authors have proposed coupled mechanochemical models to explain the mechanical contraction/relaxation using a chemical activation to describe the SMC function more realistically. (Bol et al., 2012). proposed and implemented into the framework of the finite element method a three-dimensional coupled model that describes the contractile SMC behavior setting a strain-energy function which is decomposed into a passive part (accounting for the effect of collagen and elastin fibers) and an active calcium-driven part (considering the SMC contraction). (Stalhand et al., 2008). developed a coupled model for the SMC contraction, where the cell elongation is multiplicatively decomposed in a cross-bridge deformation and a filament contraction which contribute, together with the chemical state of myosin/actin filaments, to the definition of a free energy function. (Tan and De Vita, 2015). developed a new constitutive mechanical model that takes into account the active and passive responses without introducing a kinetics chemical model, assuming that the active contractile force generated by the SMC is transmitted through the dense bodies (defined as anchoring sites) to the collagen fibers connected to them. (Murtada et al., 2017). proposed a novel multiscale mathematical model that describes arterial contractility, considering cellular, molecular, and tissue levels. This model has been validated with uniaxial and biaxial experimental data, establishing the biaxial test’s importance in identifying potential changes in vascular SMC function. Moreover, electrochemical influence on vascular SMC activity has been described in a model of steady-state nature performed by (Machingal and Ramanan, 2006), which accounts for the influence of [K^+^] [Na^+^], and [Ca^+2^] fluxes. This model works under different conditions of transmural pressure, membrane channels, and pumps blockade, and in the face of changes in ionic concentrations. The main results state a strong relation between intracellular sodium concentration [Na^+^] and SMC response.

Despite the multiple advances developed in the study of active vascular behavior, there is still no clear understanding of the parameters used in the proposed models and their quantitative influence on the stresses generated by the activation of the vascular tissue. This aspect is fundamental to establishing the parameters necessary for studying tissues of different natures or assessing how the mechanical response changes due to some disease that affects the vascular response.

This work aims to characterize the active response of a guinea pig carotid artery via the coupled mechanochemical model proposed by (Murtada et al., 2012). To this end, the isometric contraction test was performed in carotid arteries to promote the active response given by smooth muscle cell activity. At the same time, the passive response (also present in this test) was previously determined from the results reported by (Cañas et al., 2018). The parameters needed to characterize the implemented model in the context of the finite element method were found in the literature and from the experimental results of those above active and passive tests. Once the numerical implementation of the isometric contraction test was set, a sensitivity analysis was performed, identifying the most influential mechanical and chemical parameters involved in the model. The structure of this work is divided into the following parts: the experimental procedure performed in this research, along with the main aspects of both the mechanochemical model and numerical modeling of the isometric contraction test, are presented in Section 2, Section 3 describes the proposed calibration procedure of the model mechanochemical parameters and their sensitivity analysis, the obtained results are analyzed and discussed in Section 4 and, finally, the overall conclusions are drawn in Section 5.

## 2 Materials and methods

### 2.1 Material

All animal care, procedures, and experimentation were approved by the Ethics Committee of the Faculty of Medicine, from the Pontificia Universidad Católica de Chile (1130801) and Universidad de Chile (protocol CBA# 0694 FMUCH), and were conducted in accordance with the ARRIVE guidelines and the Guide for the Care and Use of Laboratory Animals published by the US National Institutes of Health (NIH Publication No. 85–23, revised 1996).

The methodology described below follows the guidelines of the study performed by (Herrera et al., 2016). The tissue analyzed in this work corresponds to the left and right segments of the carotid arteries of guinea pig fetuses. A total of 16 fetuses were collected, which size at the end of gestation allows for obtaining samples for assessments in duplicates (c.a. 90–100 gr each fetus). In this context, adult female Pirbright White guinea pigs (*Cavia porcellus*) were used for this study. All animals were housed in individual cages under standard conditions (40–45% humidity, 20–21°C, and a 12:12 h light-dark cycle), with controlled food-by-body weight intake with a commercial diet (LabDiet 5,025, Guinea Pigs, 20–30 grs/d). Pregnancies were confirmed by ultrasonography at d 20–25, where the first day with the male was considered day 0 of pregnancy (Term 67 days). Pregnant guinea pigs (*n* = 10) were subjected to aseptic surgery on gestation day 35. At 60–62 days of gestation (90% of pregnancy), the pregnant guinea pigs and their fetuses were euthanized with a maternal anesthetic overdose (Sodium Thiopentone 200 mg/kg IP, Opet, Laboratorio Chile). Once the cardio-respiratory arrest was confirmed, the fetuses were dissected and weighted. Supplementary Information about methods of obtaining, breeding, and control of the animals is detailed in SupplementaryAppendix SA.1.

The main reason for assessing the vascular mechanics in guinea pigs is related to their similarities with human gestation and physiology. A comprehensive review regarding this issue can be found in (Morrison et al., 2018). Of special interest are the parallels between human and guinea pigs’ cardiovascular maturation, placental function, and hormone profiles during gestation, which allows the study of these processes in about 60 days of pregnancy. Notably, due to the submissive behavior of guinea pigs, *ex vivo* assays can be complemented with *in vivo* characterization of fetal and maternal vascular function by Doppler in conscious animals. Additionally, we have found comparable functional changes in carotid arteries from guinea pigs (Cañas et al., 2017; Paz et al., 2019), relative to changes found in human carotid arteries (te Velde et al., 2004; Oren et al., 2004; Bjarnegard et al., 2013) from subjects with impaired fetal growth. Altogether, these data support the translational value of studying guinea pig cardiovascular samples.

### 2.2 Isometric contraction test

**FIGURE 1:**
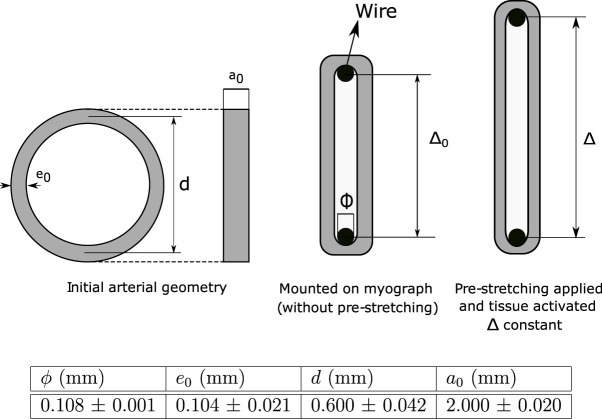
Scheme of the isometric contraction test and experimental values (± standard deviation).

The isometric contraction test was performed in carotid arteries according to the scheme shown in Figure 1. At dissection, the arteries were rapidly excised and 2.0 ± 0.02 mm-width ring samples were obtained from the proximal region of this artery (*a*_0_). The initial thickness *e*_0_ and the inner diameter of vessels were measured experimentally, where both values are used to determine the mean diameter *d*, meanwhile *ϕ* is the diameter of the wire used to clamp the artery (all values are shown in Figure 1). The experimental measurement was performed via image processing (*ImageJ* software) captured from a stereomicroscope (Motic SMZ-161) with a digital microscope camera (Moticam 2.0 MP) The ring samples were mounted on a wire myograph (610 M System Myograph multiwire, DMT), maintained at 37 °C in Krebs buffer, to determine the vasoactive responses as previously reported (Krause et al., 2016). The isometric force in response to the cumulative concentrations of KCl (16–125 mM) was registered to determine the maximal contractile force (*F*). According to the proposed constitutive model outlined in Section 2.3, the determination of this value is based on the hypothesis of complete membrane depolarization. In this work, *F* was found to be generated by a concentration of 125 mM KCl.

The initial distance between wires without pre-stretching (*Δ*_0_) can be determined as:

$$\begin{array}{c} \Delta_{0}=\pi \;\frac{d-\left(\phi +e_{0}\right)}{2}, \end{array}.$$ (1)

In addition, to a given pre-stretching *λ*, the wires separation *Δ* can be computed as:

$$\Delta =\Delta_{0}+\pi \;d/2\;\left(\lambda -1\right),$$ (2)

whose formulation considers the assumption that circumferential stretch (*λ*) of the arterial wall is dependent on wire displacement *Δ* − *Δ*_0_ (parameter measured experimentally). In addition, it is considered a perfect sliding between the hook and the arterial ring, along with a negligible bending stiffness of the arterial wall.

To determine optimal separation at which maximum active response is reached (*Δ* = *Δ*_*opt*_), an isometric contraction test was performed by setting several increasing pre-stretching values *λ*, until the contraction force levels decreased and the test ended. In the particular case of this study, seven stages were considered (*λ* = 1.11, 1.21, 1.32, 1.42, 1.53, 1.64, and 1.74) where the maximum active response was reached to *λ* = 1.64 on average.

The active Piola-Kirchhoff stress *P*_*a*_ can be obtained as the ratio between the active force registered by the wires *F* and the initial cross-sectional area:

$$P_{a}=\frac{F}{2\;a_{0}\;e_{0}},$$ (3)

This *ex-vivo* experimental method has been shown to accurately represent active responses on different biological soft tissues (Mulvany and Aalkjaer, 1990; Delaey et al., 2002). Likewise, a direct correlation of the *ex-vivo* contractile response with the biomechanical and structural properties of different blood vessels has been observed using this methodology (Cañas et al., 2017).

### 2.3 Constitutive model

In order to set a constitutive model to describe the hyperelastic material response, it is necessary to define a strain-energy function. Because the passive and active behaviors of the arterial wall are analysed, the passive (*W*_*p*_) and active (*W*_*a*_) components of the strain energy must be determined. The total strain-energy function (*W*) is assumed to be additively decomposed as (Murtada et al., 2012):

$$W=W_{p}+W_{a}.$$ (4)

These models used to describe the active and passive material behavior are described in detail below.

#### 2.3.1 Passive response

In relation to the passive mechanical response of the arterial wall, it is described according to the hyperelastic and isotropic Demiray model (Demiray, 1972), which has been used in studies of soft tissue characterization (García-Herrera et al., 2016; Cañas et al., 2018). This hyperelastic material model is defined in terms of the strain energy function (*W*_*p*_) written as:

$$W_{p}=\frac{c_{1}}{c_{2}}\exp\left(\frac{c_{2}}{2}\left(I_{1}-3\right)\right),$$ (5)

*c*_1_ and *c*_2_ is the parameters and I_1_ the first invariant of the right Cauchy-Green tensor **C**. Passive part of the tissue is associated with the fibers that surround the contractile apparatus in SMC, along with those that make up the various arterial layers, such as collagen and elastin in media and adventitia layers in the arteries.

The Demiray model parameters used in this work are those reported by (Cañas et al., 2018), which were determined in guinea pig carotid arteries of fetuses subjected to the isometric contraction test. These parameters are shown in Table 1.

**TABLE 1:** Parameters of the mechanochemical model. Symbol • denotes the paramaters determined in this study.

Passive model								
*c_1_* (kPa)	6.47	Cañas et al. (2018)	*c_2_*	2.90	Cañas et al. (2018)			

Active model								
Filament overlap parameters								
${\bar{u}}_{fs}^{\text{opt}}$	0.68	•	${\bar{x}}_{0}$	0.044	•	*λ*_opt_	1.7	•
${\bar{u}}_{e}^{\text{opt}}$	0.02	Arner (1982)	*P*_0_ (kPa)	2.45	•	*P*_opt_ (kPa)	21.22	•
Chemical parameters								
*η*(min^−1^)	21.55	Murtada et al. (2012)	ED_50_ (*μ*M)	0.37	Rembold and Murphy (1990)	*h*	1.0	•
$n_{{\bar{M}}_{p}}{|}_{t\to \infty }$	0.41	Rembold and Murphy (1988); Rembold and Murphy (1990)	*k*_*MLCK*_|_*t*→*∞*_(min^−1^)	9.79	•	*k*_*MLCP*_(min^−1^)	14.10	•
*k*_3_ (min^−1^)	8.0	•	*k*_4_ (min^−1^)	0.05	Murtada et al. (2012)	*k*_7_ (min^−1^)	0.002	Murtada et al. (2012)
Mechanical parameters								
*μ*_*a*_ (kPa)	2,926.6	•	*β*_1_ (min^−1^)	0.50	Murtada et al. (2012)	*β*_2_ (min^−1^)	0.125	Murtada et al. (2012)
*P*_*LBC*_ (kPa)	118	Dillon et al. (1981)	*κ*_*AMp*_ (kPa)	147.5	•	*κ*_*AM*_ (kPa)	61.1	Murtada et al. (2012)
*α* (kPa)	26.7	Murtada et al. (2012)						

#### 2.3.2 Active response

According to (Holzapfel, 2000), the active strain-energy function (*W*_*a*_) is stated as:

$$W_{a}=\frac{\mu_{a}{\bar{L}}_{o}}{2}\left(n_{AM_{p}}+n_{AM}\right){\left(\lambda -{\bar{u}}_{fs}-1\right)}^{2},$$ (6)

where *μ*_*a*_ is a stiffness parameter, ${\bar{L}}_{o}$ and ${\bar{u}}_{fs}$ are parameters related to the sliding mechanism which governs the active response to cellular level, meanwhile $n_{AM_{p}}$ and *n*_*AM*_ are mass fractions that account contraction activity states. Details related to the mechanism of active contraction are expanded in Supplementary Appendix SA.2, in which the origin of the aforementioned parameters is specified. Note that Expression six is particularized to the case of the 1D uniaxial ring tensile test for calcium-driven SMC contraction. A general stress-stretch relationship is described below.

#### 2.3.3 Generalized stress tensor

The Second Piola Kirchhoff stress tensor **S**, extended to a general case, is given by:

$$S=F^{-1}P=2\frac{\partial \left(W_{a}+W_{p}\right)\left(C\right)}{\partial C}-pC^{-1},$$ (7)

$$\begin{array}{cc} S & =c_{1}\exp\left[\frac{c_{2}}{2}\left(C:I-3\right)\right]I+\mu_{a}{\bar{L}}_{o}\left(n_{AM_{p}}+n_{AM}\right)\left(\sqrt{I_{4}}-{\bar{u}}_{fs}-1\right)\frac{1}{\sqrt{I_{4}}}{\hat{a}}_{0}⊗{\hat{a}}_{0}-pC^{-1} \end{array},$$ (8)

where **F** is the deformation gradient, **P** the first Piola Kirchhoff stress tensor, ${\hat{a}}_{0}$ defines the SMC orientation in the arterial ring (considered circumferentially in this work), I_4_ represents the fourth invariant of the right Cauchy Green strain tensor **C** ( $I_{4}={\hat{a}}_{0}\cdot C\cdot {\hat{a}}_{0}$ , where I_4_ = *λ* ^2^ in the particular 1D case), and p is the Lagrange multiplier which enforces the incompressibility, commonly assumed on soft tissues. Expression eight is considered in the 3D numerical simulations shown below.

### 2.4 Numerical simulation

In order to simulate the isometric contraction test of a ring-shaped arterial tissue, the mechanochemical formulation detailed in Section 2.3 was implemented in an in-house finite element (FE) code (García-Herrera and Celentano, 2013). Prior to the simulation $n_{AM_{p}}$ , *n*_*AM*_ and ${\bar{u}}_{fs}$ , defined in Eq. 6, are determined by ordinary differential equations (ODE) (see Supplementary Appendix SA.2) *via* an explicit Euler method, whose general form is expressed by:

$$y_{n+1}=y_{n}+sf\left(t_{n},y_{n}\right),$$ (9)

being *y* the parameter to be determined to a particular step *n* and to the next *n* + 1, s the step size and $f\left(t_{n},y_{n}\right)$ a characteristic function according to the ODE to be solved.

**FIGURE 2:**
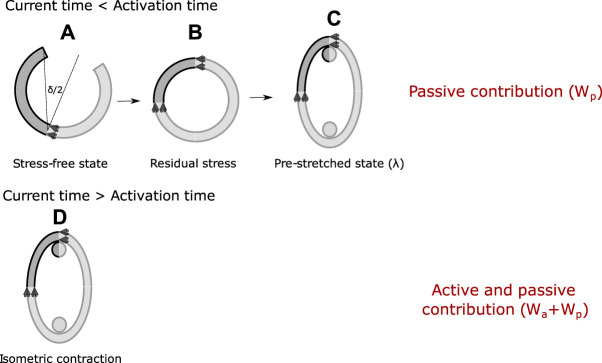
Numerical procedure followed in the simulation of the isometric contraction test. The dark-gray shaded areas represent the geometry considered for the simulation. Symmetry along thickness was considered in all cases, with the corresponding boundary condition.

Figure 2 shows the full numerical procedure carried out, which involves the presence of circumferential residual stress (A-B) and those generated by the initial pre-stretching applied *λ* (B-C). Specifically, the residual stress field is computed via ring closure procedure (results reported by (Cañas et al., 2018)), which considers that an opened configuration (experimental value of *δ* = 86.10° ± 4.01°) is the stress-free state (García-Herrera et al., 2016). This simulation was carried out considering one quarter opened ring and the corresponding symmetry constraints (see A from Figure 2). In addition, numerical simulation of initial pre-stretching *λ* was performed taking one-eighth of the closed ring with the respective symmetries (B and C from Figure 2). Both types of stresses mentioned previously are determined through the hyperelastic passive contribution (described by *W*_*p*_ in Expression 5).

**FIGURE 3:**
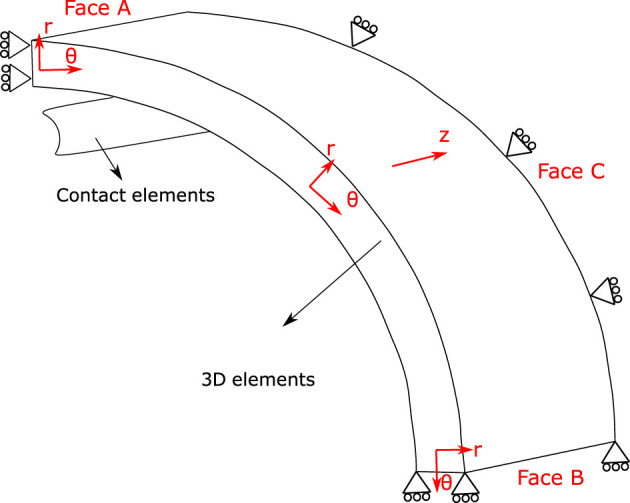
Geometry of the isometric contraction test considered in the numerical simulation. Cylindrical coordinates define *θ* axis as circumferential direction, *r* axis as radial direction and *z* axis as axial direction. Boundary conditions: one-eighth of the ring is considered, by constraining the displacement on the *θ* axis in the Face A and B, along with the axial movement in the Face C.

On the other hand, stress values from numerical simulation of isometric contraction test (defined by an activation time) are determined by the hyperelastic active and passive contributions, described by the sum of *W*_*a*_ + *W*_*p*_ (D from Figure 2). Due to symmetry conditions, as for B, only one eighth of the ring is considered with boundary conditions depicted in Figure 3, by constraining the displacement on the *θ* axis in the Face A and B, along with the axial movement in the Face C.

The finite element mesh used to discretize the domain is composed of nearly 3,800 8-node hexaedral solid elements while the wire is modelled with rigid frictionless contact surface elements, through an adequate contact pressure function (P_n_), which is formulated to increase quadratically with the penetration (g_n_) of the surfaces considered:

$$P_{\text{n}}=E_{\text{n0}}g_{\text{n}}+E_{\text{n}}g_{\text{n}}^{2},$$ (10)

being E_n0_ and E_n_ are constants that control the penetration. Finally, the incompressibility condition characteristic of hyperelastic material was incorporated by an appropriate penalty function (*κ*F_vol_) of the volumetric component of Second Piola-Kirchhoff stress tensor **S**_vol_ (Holzapfel, 2000):

$$S_{\text{vol}}=\kappa F_{\text{vol}}JC^{-1},$$ (11)

where *κ* is an incompressibility parameter (set to a level of 10^3^–10^4^ times *c*_1_), J is the determinant of the deformation gradient **F**, and F_vol_ corresponds to the volumetric function, defined in this case as J – 1.

### 2.5 Sensitivity analysis

Through the numerical simulation of the isometric contraction test, the most preponderant chemical and mechanical parameters of the model were determined through a sensitivity analysis in order to quantify the active response of the guinea pig carotid artery. According to the parameters displayed in Supplementary Appendix SA.2, the analysis was focused on the chemical parameters *h* (Expression 14), *k*_3_, *k*_4_ and *k*_7_ (Expression 13) all of them related to the rate of evolution of activation mechanisms at the cellular level; and mechanical parameters *κ*_*AM*_ and *κ*_*AMp*_, both related to the load capacity on activation process (Expression 22). In all cases, three values were considered for each parameter taking as intermediate values those set by (Murtada et al., 2012). Once sensitivity was performed, the most influential parameter on the active response was stated (Section 3.2), through the definition of a sensitivity index (**si**), which is assessed by:

$$si=\sqrt{{\left(\frac{P_{a+}-P_{a0}}{P_{a0}}\right)}^{2}+{\left(\frac{P_{a-}-P_{a0}}{P_{a0}}\right)}^{2}},$$ (12)

where *P*_*a*0_ corresponds to the stationary active stress at the intermediate value of the parameter under study, whereas *P*_*a*+_ and *P*_*a*−_ are the active stresses obtained to a positive and negative variation of this parameter, respectively. The high value of **si** determines the most sensitive parameter, which is numerically fitted, using the experimental data obtained from this study (Section 3.3).

## 3 Results

### 3.1 Axial stress-stretch curve

Table 1 summarizes the numerical values of the parameters associated to the mechanochemical model implemented in this work. Some parameters are based on previous works and others are obtained from this research. It should be noted that the parameters cited in Table 1 are obtained directly from these references and they are not the result of the study presented, whereas the rest of the parameters are determined in this study, through the methodology exposed in Section 2. Details about the origin of all of them can be found in Supplementary Appendix SA.2.

**FIGURE 4:**
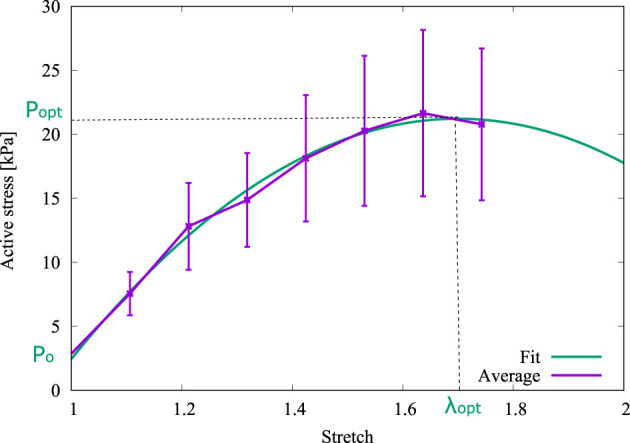
Derivation of *P*_0_, *P*_opt_ and *λ*_opt_ from the fitted curve.

The active stress (*P*_*a*_)–stretch curve is plotted in Figure 4, which was obtained by subtracting the passive contribution determined in a tensile test on an arterial ring sample (Cañas et al., 2018). This fitting was obtained from the experimental data through a second order polynomial regression via normalized root-mean-square deviation (NRMSD), with the experimental information available. As it is seen in Figure 4, *P*_0_, *P*_opt,_ and *λ*_opt_ were determined through a numerical fitting of this curve. The resulting values are shown in Table 1.

Owing to the parameters used by (Murtada et al., 2012) were obtained from experimental results of pig carotid arteries whereas the present research is based on the biomechanical results from guinea pig carotid arteries, some parameters of the mechanochemical model must be fitted from the isometric contraction test. From Expression 2 with the average dimensions of Figure 1 and setting *λ* = *λ*_opt_, the optimal wire separation *Δ* = *Δ*_opt_ = 1.215 mm has been be determined. Then, other values of the mechanochemical model parameters can be set: the optimal fiber sliding ${\bar{u}}_{fs}^{\text{opt}}$ was obtained according to Expression 17, the relative filament overlap ${\bar{x}}_{0}$ was determined from Expression 19 and, finally, the stiffness constant *μ*_*a*_ was computed from the optimal values evaluated with Expression 20. These values are indicated in Table 1.

### 3.2 Sensitivity analysis

**FIGURE 5:**
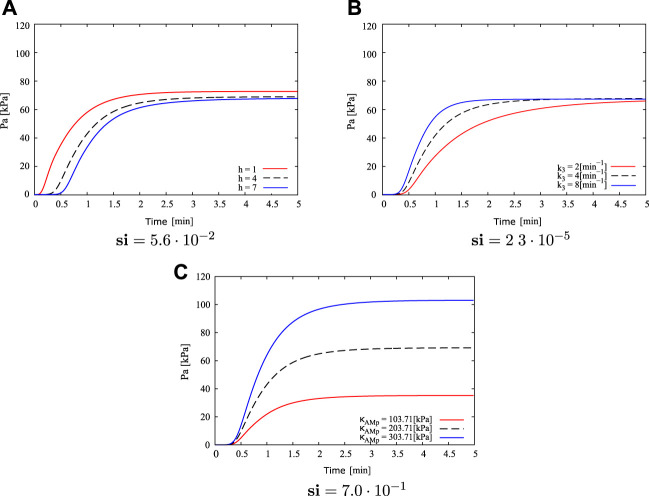
Sensitivity analysis for parameters: **(A)** *h*, **(B)** *k*_3_ and **(C)** *κ*_*AMp*_, regarding to active stress *P*_*a*_, and its corresponding **si** value.

Figure 5 shows the results of the most influential parameters with respect to the active stress *P*_*a*_ evolution: *h*, *k*_3_ and *κ*_*AMp*_. From Figure 5A, when the parameter *h* is diminished (*h* = 1) in relation to the reference value (*h* = 4), the rate to reach the steady-state active stress value is higher (it takes around 3 min) and the activation time (time elapsed since the specimen already has been activated) is lower (0.15 min approximately). By incrementing this parameter (*h* = 7), longer times are required to reach the maximum in active stress, where in this case it occurs approximately 4–5 min after the activation begins. In addition, the activation time increases, reaching 0.4 min. Concerning the steady-state stress value, three curves do not exhibit noticeable differences, where in all cases this value varies between 67 and 73 kPa. Figure 5B shows that variation in *k*_3_ value only affects the time to reach the maximum constant value. In cases where this parameter increases (*k*_3_ = 8min^−1^), transient velocity is higher contrasting active response in cases with lower values (*k*_3_ = 2min^−1^). In the first case, steady-state active stress is reached to 1.5 min approximately, whereas in latter case it is reached around 4.5 min. Both the steady-state active stress and activation time are practically the same in three cases studied (around 68 kPa and 0.25 min respectively). In the same way as for the parameter *k*_3_, Figure 5C shows that the mechanical parameter *κ*_*AMp*_ only affects one specific characteristic of the active stress evolution, which is related to the maximum value of *P*_*a*_ reached at the steady-state condition, where an increment in *κ*_*AMp*_ triggers a high active strees with respect to lower values of *κ*_*AMp*_. Specifically, when *κ*_*AMp*_ = 303.71 kPa the steady-state *P*_*a*_ value is around 105 kPa, while for the reference value *κ*_*AMp*_ = 203.71 kPa the maximum *P*_*a*_ is 70 kPa, and for the lowest parameter value studied *κ*_*AMp*_ = 103.71 kPa the maximum active response reaches 35 kPa. In relation to the activation time and active stress rate, the curves illustrate that variations of the *κ*_*AMp*_ parameter do not induce a change of these values. Comparing these cases, it is possible to determine that *κ*_*AMp*_ has the most noticeable influence on the maximum active stress reached, while changes in the *h* and *k*_3_ values greatly affect the active stress rate. In this way, the sensitivity index (**si**) stated in Eq. 12 and displayed in Figure 5, reflects that *κ*_*AMp*_ has a higher influence in the active stationary response. Therefore, this is the most critical parameter for fitting the constitutive model, and its determination will be performed below.

### 3.3 Determination of parameter *κ*_*AMp*_ *via* numerical fitting

**FIGURE 6:**
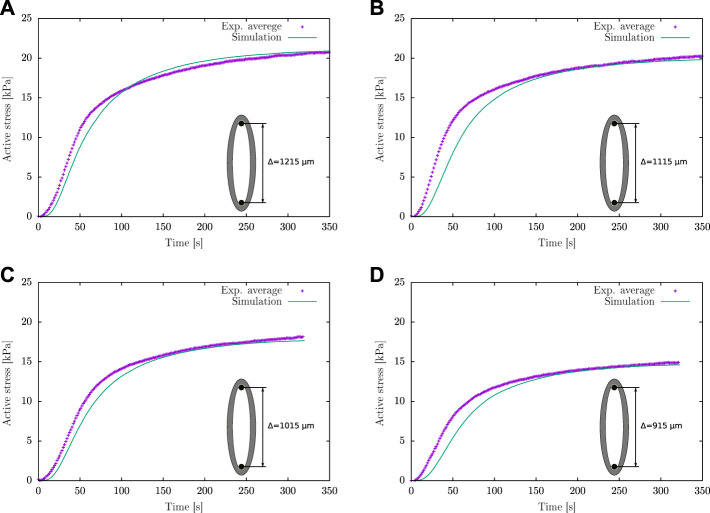
Active stress for different wire separations in the isometric contraction test: **(A)** *Δ* = *Δ*_opt_ = 1,215 *μ*m, **(B)** *Δ* = 1,115 *μ*m, **(C)** *Δ* = 1,015 *μ*m and **(D)** *Δ* = 915 *μ*m.

Based on the comparison of the experimental information considered in (Murtada et al., 2012), it is determined that the active response of the guinea pig carotid artery used in the current study presents a higher activation rate in the transient state at a lower contraction force in the steady state. Therefore a fitting of *κ*_*AMp*_ parameter is performed considering *h* = 1 and *k*_3_ = 8 (Figure 5; Table 1), which represent the conditions mentioned previously. Taking as reference the *κ*_*AMp*_ value that generates the lower active stress *P*_*a*_ (*κ*_*AMp*_ = 103.71 kPa), numerical simulations of the isometric contraction test were performed for different wire separations varying this parameter until a minimum NRMSD is achieved. Table 2 exhibits the two best fits for the *κ*_*AMp*_ value, which are denoted by Sim 1 and Sim 2. Four different conditions related to the wire separation in the isometric contraction test were studied (i.e., from 915 to 1,215 *μm*, which corresponds to the optimal wire separation), which are plotted in Figure 6. According to the average NRMSD of the analysed stretching conditions, the best overall approximation to the *κ*_*AMp*_ value is 147.5 kPa given by the Sim 2 cases, even though in one specific case (wire separation of 1,215 *μ*m) the simulation with *κ*_*AMp*_ = 145 kPa exhibits a lower NRMSD. Figure 6 shows the best fit obtained by the results of numerical simulation to the experimental average curves for the four wire separations, where the maximum active stress is reached when the wires are separated by the optimal distance *Δ*_opt_ (Figure 6A). Finally, from this sensitivity analysis, the numerical value adopted for the *κ*_*AMp*_ parameter is shown in Table 2.

**TABLE 2:** Normalized root-mean-square deviation for different *κ*_*AMp*_ values.

Wires dist	1,215 (*μ*m)		1,115 (*μ*m)		1,015 (*μ*m)		915 (*μ*m)		Average	
	Sim 1	Sim 2	Sim 1	Sim 2	Sim 1	Sim 2	Sim 1	Sim 2	Sim 1	Sim 2
*κ*_*AMp*_ (kPa)	145	147.5	145	147.5	145	147.5	145	147.5	**145**	**147.5**
NRMSD	3.14	3.23	8.32	7.88	5.03	4.14	6.89	6.49	**5.85**	**5.43**

### 3.4 Active stress and stretch profiles along the tissue thickness

**FIGURE 7:**
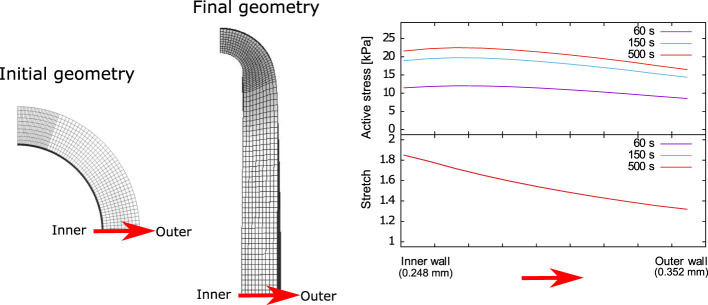
Active stress and stretch variation along the carotid artery wall thickness.

From the numerical simulation of the isometric contraction test, the spatial variation of both the active stress and stretch along the tissue wall thickness are plotted in Figure 7 at different times, away from the contact zone between the artery and the wire. It is seen that the maximum active stress value develops at approximately 0.03 mm from inner wall. This trend is the same at all times, where the maximum *P*_*a*_ value at the steady-state condition (time 500 s) is 22.2 kPa approximately. Moreover, the stretch profiles are found to remain unchanged along time (i.e., the three curves are overlapped), with decreasing values from the inner to the outer zones of the artery wall, from 1.9 to 1.3 approximately.

## 4 Discussion

It should be noted that the active stress describes the mechanical response *P*_*a*_, which is the only experimental variable measured through active force *F* (Eqs 2, 3. Therefore, it will be used as a comparison variable for the different aspects studied. As can be seen in the model description in Supplementary Appendix SA.2, *h* plays a direct influence in the phosphorylation activity through *k*_*MLCK*_ (Expression 14), while the dephosphorylation activity, described by means of *k*_*MLCP*_, depends in turn on *k*_*MLCK*_ (Expression 16). Small *h* values trigger higher *k*_*MLCK*_ and *k*_*MLCP*_ values; however, a greater impact of *h* parameter is seen on *k*_*MLCK*_ than in *k*_*MLCP*_. Moreover, according to the latch-state model and the definition of driving and active stresses (*P*_*c*_ and *P*_*a*_ in Expressions 22 and 20 respectively), the *n*_*AMp*_ and *n*_*AM*_ fractions are taken into account due to their influence on the active response (*P*_*a*_), while the driving stress *P*_*c*_ depends only on *n*_*AMp*_ according to the contractile conditions studied in this work $\left(P_{c}^{\mathrm{cntr}}\right)$ . Since this latter variable is responsible to set the maximum load-bearing capacity, the active stress *P*_*a*_ will be limited by *P*_*c*_. As low *h* values promote phosphorylated states, the fraction *n*_*AMp*_ increases and, consequently, the driving stress *P*_*c*_ is greater. Therefore, the steady-state active stress *P*_*a*_ is also higher under this condition. In relation to the active stress evolution (Figure 5A), it is faster for small *h* values, condition that triggers high *k*_*MLCK*_ values, as mentioned previously. By definition, *k*_*MLCK*_ corresponds to the rate of change from a dephosphorylated state to a phosphorylated one. Then, while this value is high, the active response will change faster. The parameter *k*_3_ regulates the rate of change between detached and attached phosphorylated myosin cross-bridges, that is, from $n_{M_{p}}$ to $n_{AM_{p}}$ , respectively. As shown in Figure 5B, when *k*_3_ is higher, maximum active stress *P*_*a*_ is quickly reached; meanwhile, at low *k*_3_ values, maximum active stress is reached in a long time. However, in both cases, the steady-state active and driving stress are the same. A possible explanation to this phenomenon is based on the fact that *k*_3_ only influences on how fast it changes from a detached-phosphorylated state *n*_*Mp*_, which does not account neither *P*_*a*_ nor *P*_*c*_, to an attached-phosphorylated state *n*_*AMp*_, where there is an influence on *P*_*a*_ and *P*_*c*_. Then, both fractions will reach steady-state values in a stationary state, and the active response *P*_*a*_ will have the same values in all analyzed cases. This response is because changes in *k*_3_ do not affect the steady-state fractions of different states of the latch-state model. This can be observed when a steady-state condition is reached, in which no temporary changes of any variables exist. It is worth stating that changes in all other activity values influence the steady-state fractions. In addition, the most dominant mechanical parameter is *κ*_*AMp*_, which is associated directly with the driving stress *P*_*c*_. The parameter *κ*_*AMp*_ is also related to the force exerted in a power-stroke by a cross-bridge. It is considered when the fibers are in contraction or relaxation states. Therefore, *P*_*c*_ limits the upper range of the active stress *P*_*a*_ value that can be reached, which is lower when *κ*_*AMp*_ is reduced (Figure 5C). The change in *P*_*a*_ is proportional to the variation in *κ*_*AMp*_ concerning the referential value.

According to the numerical and experimental results of the isometric contraction test (Figure 6), they exhibit a good fit in the steady-state condition, where in all cases to times beyond 150 s, there is a better correlation than in the transient state, in which significant differences between both results are presented. This could be explained due to the mechanochemical model nature because some variables like the dephosphorylated activity *k*_*MLCP*_ depend on the steady-state fractions of detached cross-bridges and phosphorylated activity (see Expression 16). In addition, the filament overlap as a function of the filament sliding (Expression 17) comes from a polynomial fit to the true behavior of the actin sliding. Both curves coincide specifically for the case where the steady-state is reached ${\bar{u}}_{fs}={\bar{u}}_{fs}^{\text{opt}}$ . Therefore, this model does not consider some aspects of the transient state, thus prioritizing a good fit in the steady-state condition. To verify the consistency of the results, the distribution of the active stress *P*_*a*_ through the ring specimen thickness is shown (Figure 7). This variable reaches the maximum levels near the inner wall region (approximately 0.02 mm), decreasing towards the outer diameter. Regarding the *P*_*a*_ evolution, the maximum values of the active stress are given once the steady-state is reached, the time at which the maximum load-bearing capacity of each CU has been reached. About the active stress and its effect on the stretch values along with the thickness, it is possible to appreciate a good correlation between the optimal stretching *λ*_opt_ and the maximum values of the active stress *P*_opt_, when the results obtained from the simulation are compared (Figures 4, 7). Specifically, in the simulation of the isometric contraction test, the stretch value is close to 1.7, that is, similar to the one determined as optimal stretching *λ*_opt_ = 1.7. In contrast, the maximum stress value from the numerical results is close to 22 kPa (*P*_opt_ = 21.22 kPa), which is consistent with the theory presented. It is worth mentioning that the numerical results shown previously incorporate circumferential residual stress owing to the ring specimen is not in a free-stress state, thus providing more realistic and reliable results. According to the studied case in this work, the effect of this residual stress could not be comparable with the active stress reached since the higher principal residual stress in the carotid artery of guinea pig is around 1 kPa. In comparison, the active stress peaks are in the order of 20 kPa. The relevance of the trans-mural stress field exhibited is questionable from a physiological point of view due to the non-homogeneity of smooth muscle cell (SMC) density across the artery wall (Holzapfel, 2000). This aspect is not accounted for in the model, which assumes the same distribution of SMC by each arterial layer (Eq. 4) (Rachev and Hayashi, 1999). Despite this, the overall active stress values displayed do have a physiological meaning because model parameters are fitted to experimental data as shown in Figure 6.

Special attention should be given to the methodology aspects (Section 2). Specifically, it is important to remark that the consideration of the circumferential strain (*λ*) referred on Expressions 1 and 2 takes as the main assumption that *λ* is dependent on wire displacement (*Δ* − *Δ*_0_), whose value is experimentally measured. Additional aspects consider a perfect sliding between the hook and the arterial ring and that the bending stiffness of the arterial wall is negligible. On the other hand, *W*_*p*_, corresponding to the definition of passive strain energy function given by the Demiray model (Expression 5), could be defined in terms of alternative models, such as Holzapfel-Gasser-Ogden (Gasser et al., 2006). However, due to the anisotropic nature of this model, it requires mechanical response information in more than one direction. Due to the experimental limitations of arterial size, it is impossible to perform in guinea pig carotid arteries. The future redefinition of *W*_*p*_ would not significantly affect the experimental procedure carried out in this work.

To set the scope of this study, different shortcomings are identified. Referred to the experimental part, although the ring isometric contraction test studies the active contraction of an arterial segment, this is analyzed in a uniaxial tension state. In this sense, the main limitation is that such a configuration does not mimic the *in-vivo* condition, so the mechanisms of arterial tissue activation under the condition studied in this work are not necessarily the same as in the *in-vivo* state. Ideally, the experimental procedure should consider pressurization and axial stretch on the artery duct (Murtada et al., 2017). According to the study performed by (Reynolds et al., 2020) cell alignment, and consequently the active contractility, which is dependent on the constrained condition (uniaxial or biaxial), may be modified in the face of dynamic loads and intracellular calcium concentration [Ca^2+^]. Focusing the discussion on the limitation of the mechanochemical model presented, an aspect being highlighted is that the current implementation considers that the SMC remain constant in orientation, concentration, and functionality in response to the external conditions applied to the tissue under study. However, there is evidence in the literature that, at the cellular level, mechanical microenvironment signals are sensed by cells, regulating the genetic expression and affecting the properties of the nucleus (Alisafaei et al., 2019). To study the *in vivo* state, the formulation detailed in this work should account for the differences between the evolution of the *in vivo* intracellular calcium concentration [Ca^2+^] and the conditions experimentally imposed in the isometric contraction test. According to work carried out by (Brekke et al., 2006), the [Ca^2+^] concentration curve in an *in-vivo* state exhibits an alternating behavior, without the presence of a steady-state regime, unlike what can be deduced from Expression 15. This condition triggers a time-dependent behavior of the activity for the different states *k*_*i*_, and, consequently, the different fractions of the latch state model (*n*_*i*_) will also change (both categories of parameters referred to in Expression 13). On the other hand, the *in vivo* condition would be related to a variable pressurization condition fluctuating between systolic and diastolic values. In this case, at each time step, the circumferential elongation (denominated as *λ* in this work) must be identified to quantify the active stress *P*_*a*_, dependent on the overlapping between the actin and myosin fibers ${\bar{u}}_{fs}$ (with the assumption that SMC are circumferentially oriented).

It is essential to emphasize that previous works in the area of active models describe different features related to the multi-scale activation mechanisms and determine the parameters that account for these processes. Several models have been proposed to explain the activation mechanisms of the arterial wall; there are many aspects in common with the considerations of the model detailed in Supplementary Appendix SA.2, such as the sliding filament theory (Gestrelius and Borgstrom, 1986; Murtada et al., 2017), the kinetic of myosin phosphorylation (Stalhand et al., 2008; Murtada et al., 2010) and active calcium-driven of SMC contraction (Bol et al., 2012; Murtada et al., 2016). Given this consideration, the model formulated by (Murtada et al., 2012) appears as the most adequate to characterize the active-passive response of the studied artery due to its 1-D formulation, given the small dimensions of guinea pig carotid arteries, where only experimental information of isometric contraction in the circumferential direction is available. There are more sophisticated models in literature which account for extra consideration in the contraction mechanism, such as the non-alignment of SMC in circumferential direction (Murtada et al., 2017), or the existence of active collagen fibers linked with SMC (Tan and De Vita, 2015). In these cases, a more significant number of parameters must be defined; therefore, additional tests are necessary to perform the model parameters fitting (e.g., biaxial isometric contraction test or adding the isotonic contraction test).

## 5 Conclusion

An extensive analysis using a mechanochemical model aimed at describing the active behavior of the vascular tissue has been performed in this work, focusing its application on experimental results obtained from a carotid artery of guinea pig fetuses. To this end, an experimental procedure considering the isometric contraction test was carried out, which provided information about the mechanical response of arterial tissue subjected to chemical activation. In particular, the optimal stress (*P*_opt_) was found to reach 22 kPa to an optimal stretch (*λ*_opt_) of 1.7. The parameters involved in the mechanochemical model, whose implementation was performed in the context of the finite element method, were obtained through experimental results and literature review. Numerical implementation of the isometric contraction test showed a good correlation with the experimental results, especially the active stress under stationary conditions. Furthermore, the numerical simulation provides complementary information related to the stress distribution through the arterial wall, which agrees with the stress/strain relation observed from the experimental results. According to the results, one of the main shortcomings of the model is related to the fact that the studied model does not respond well when comparing the transient evolution of the studied variables. Therefore, the future investigation should focus on implementing an improved model that incorporates a realistic behavior of the transient response of the tissue. In this sense, combining viscoelastic and anisotropy effects in the passive part of the model could give a complete approximation to the arterial mechanical response, for which a set of experimental tests are required.

## Data Availability

The original contributions presented in the study are included in the article/Supplementary Material, further inquiries can be directed to the corresponding author.
