# Supplementary material for: Characterization of the active response of a guinea pig carotid artery
Source: Front Bioeng Biotechnol. 2022 Aug 26;10:924019. doi: 10.3389/fbioe.2022.924019 (PMC9458959; doi:10.3389/fbioe.2022.924019)
Supplement: Supplementary file 1 [file DataSheet1.pdf]

A Appendix

A.1 Details about experimental design

The animals included in this work belong to a control group of a protocol for inducing fetal growth restriction. A complete description of the surgery performed at 35 days of gestation can be found in Herrera et al., 2016. Briefly, under general anesthesia (ketamine 60 mg/kg, xylazine 4 mg/kg, and atropine 0.1 mg/kg IM) an infra-umbilical midline laparotomy was performed, exposing the gravid uterus. In the FGR group, ameroid constrictors (COR-2.00-SS; NW Research Instruments, Inc, USA) were placed bilaterally around the base of each uterine artery. The abdominal wall and skin were then sutured in layers with absorbable sutures (Vicryl 4/0; Ethicon, USA). Finally, surgical staples (AutoSuture; Covidien, Dominican Republic) were installed in the skin. As part of this procedure, animals received analgesia (carprofen 4 mg/kg SC) and prophylactic antibiotic (20 mg oxytetracycline/kg SC) treatments. The skin staples were removed 7-8 days after surgery. The control group, which is the subjects specifically reported in this study, underwent the same surgical procedure, but without placement of the ameroid constrictors. Guinea pigs were obtained at the Instituto de Salud Pública, Chile, which is the local provider of experimentation animals, authorized by the Chilean Ministry of Health. Female guinea pigs were between 5- to 6-month-old, meanwhile, males were between 5- to 10-month-old at mating times. Guinea pig sows were in single cages and periodically examined by a trained veterinarian to follow their estrous cycle. Females were placed with a male for 1 week after full perforation of the vaginal membrane was noted. Ultrasound and doppler assessment (mentioned in Section 2.1) were performed using a Sonovet R3, Samsung Medison, equipped with a Medison LN5-12 Linear [5-12 MHz] transduced for small animal and superficial imaging.

A.2 Mechanochemical model

The model used to describe the active mechanical response of the arterial wall is based on the mechanochemical model proposed by Murtada et al., 2012, which considers phosphorylated/dephosphor
states of cross-bridges due to increment/decrement of intracellular [Ca^{+2}] level (chemical contribution) and sliding of actin and myosin fibers, both mechanisms that finally triggers the SMC contraction (mechanical contribution). Different variables of the active behavior defined below are set in the reference space known as a contractile unit (CU), composed by actin and myosin filaments in addition to myosin cross bridges, where each CU is separated by a dense body; see Figure 1. In order to explain as clearly as possible the different variables that compose the mechanochemical model, sub-sections have been created to define its chemical and mechanical contributions and that related to the mechanism of fibre contraction.

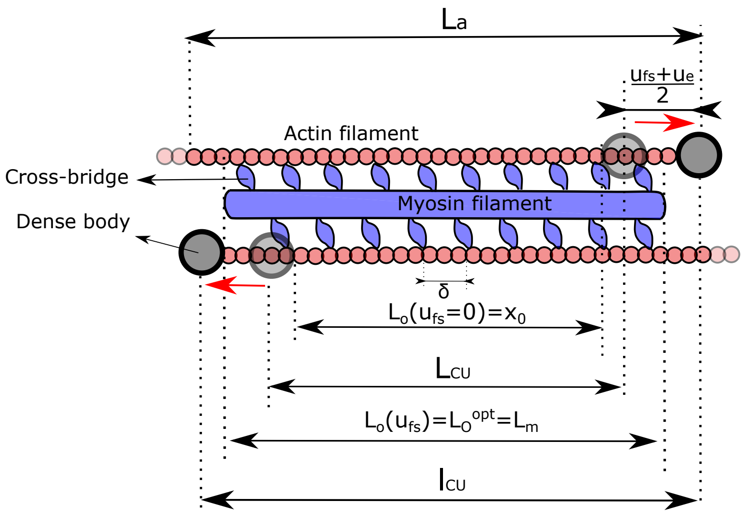

Figure 1 Contractile unit (CU) scheme.

Latch-state model

SMC contraction is described according to kinetics latch-state model proposed by Hai and Murphy, 1988, which sets four possible states in which calcium dependent myosin phosphorylation

is the only existing mechanism in the contraction/relaxation process (Murtada et al., 2012). Figure 2 shows the main aspects of this model. The initial state M is associated to dephosphorylated and detached myosin head from actin, where a new ATP molecule attaches on the cross-bridge myosin. In the state M_p, the myosin head is phosphorylated by means of the ATP hydrolysis, generating ADP and inorganic phosphate (P). This process is possible due to the presence of $Ca^{+2} - CaM$ complex which activates the MLCK enzyme. In the state AM_p, the highly energyzed myosin head is attached to the actin filament. Between M_p and AM_p states, the cross-bridge cycle occurs, where from the AM_p state P is released and a power stroke allows the sliding of the actin filament, releasing in turn an ADP molecule. Then, a new ATP attaches the myosin head detaching it from the actin filament. Finally, this cycle is repeated. On the other hand, the state AM defines the latch-state, where the dephosphorylated head remains attached to the actin, aspect that is included in the model due to experimental observations for high levels of tension at low levels of phosphorylation (Murtada et al., 2012). All states are reversible, except the transition from the state AM to M. The rate of transition between each state is shown in Figure 2, where k_{MLCK} corresponds to a shift from a dephosphorylated to a phosphorylated state under both attached and detached conditions such that its value is related to the MLCK activity. In the same way, k_{MLCP} is associated with the rate of transition from a phosphorylated to a dephosphorylated state, representing the MLCP activity. k_3 and k_4 respectively represent attachment and detachment of the cross-bridge cycle, while k_7 is the rate of latch-bridge detachment.

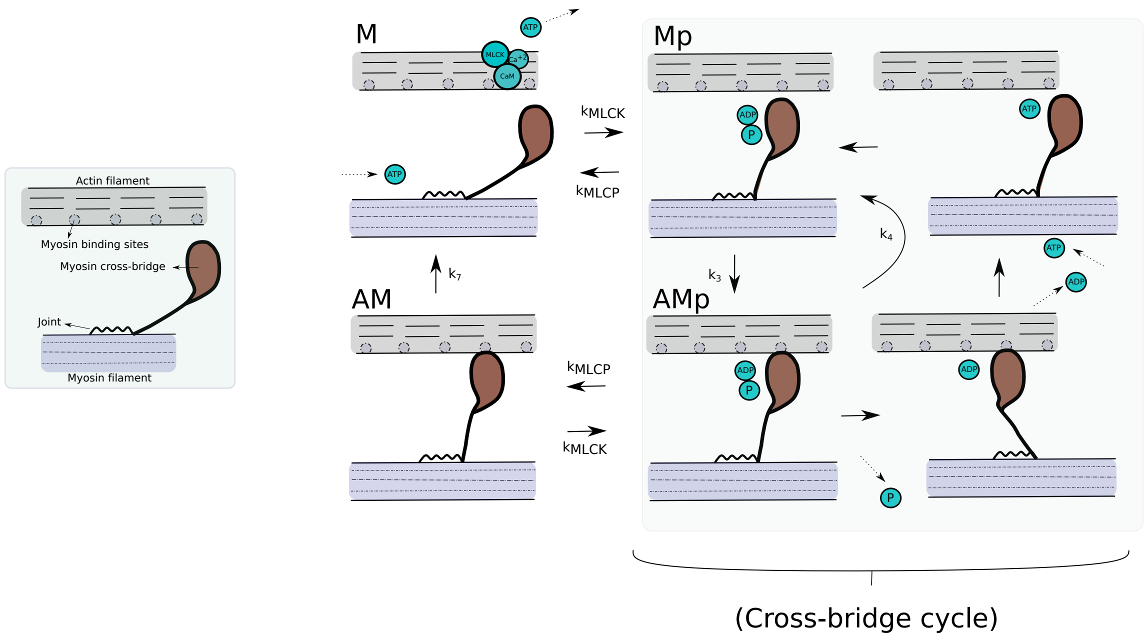

Figure 2 Latch-state model scheme.

The kinetic model is represented by a set of differential equation written as:

$$\begin{bmatrix} \dot{n}_M \\ \dot{n}_{M_p} \\ \dot{n}_{AM_p} \\ \dot{n}_{AM} \end{bmatrix} = \begin{bmatrix} -k_{MLCK} & k_{MLCP} & 0 & k_7 \\ k_{MLCK} & -(k_{MLCP}+k_3) & k_4 & 0 \\ 0 & k_3 & -(k_{MLCP}+k_4) & k_{MLCK} \\ 0 & 0 & k_{MLCP} & -(k_{MLCP}+k_7) \end{bmatrix} \begin{bmatrix} n_M \\ n_{M_p} \\ n_{AM_p} \\ n_{AM} \end{bmatrix} \quad (1)$$

where n_M, n_{M_p}, n_{AM_p} and n_{AM} indicate the fractions of each possible state of the latch-state model described previously (the sum of all fractions must be equal to 1) and $\dot{n}_M$, $\dot{n}_{M_p}$, $\dot{n}_{AM_p}$ and $\dot{n}_{AM}$ are their corresponding time derivatives. In adittion, it is assumed as initial condition that all the cross-bridges are dephosphorylated and detached ($n_M = 1$ and $n_{M_p} = n_{AM_p} = n_{AM} = 0$). From Murtada et al., 2012, the relation between the rate of phosphorylation k_{MLCK} and the intracellular calcium concentration evolution $[\mathrm{Ca}^{+2}](t)$ have been determined:

$$k_{MLCK} = \eta \, \frac{[\mathrm{Ca}^{2+}]_i^h}{[\mathrm{Ca}^{2+}]_i^h + (\mathrm{ED}_{50})^h} \tag{2}$$

$$[\mathrm{Ca}^{2+}]_i(t) = \exp\left(-1.31(t^{1.25} - 4.53)\right)\sin(t + 11.96) + 308.0 \tag{3}$$

where η and h are fitting parameters and ED_{50} is a constant value related to the half-activation for $[\mathrm{Ca}^{+2}]_i$ to MLCK at a constant level of CaM. In the specific case of uniaxial ring test, the membrane depolarization of SMC is independent of diffusion-delay phenomenon, due to in the experiment the depolarization-inducing agent (KCl) comes in direct contact with some SMC (Murtada et al., 2017). The temporal variation of $[\mathrm{Ca}^{+2}]_i$, expressed by Expression 3, was fitted by an appropiate function according to the experimental data available from Murtada et al., 2012.

To determine the k_{MLCP} value, it is stated that temporal changes of the phosphorylation and dephosphorylation processes do not exist in the steady-state condition, i.e., $\dot{n}_{\bar{M}} = \dot{n}_{\bar{M}_p} = 0$. Therefore, a constant k_{MLCP} value is determined, depending on the steady-state condition ($t \to \infty$), as:

$$k_{MLCP} = k_{MLCK}|_{t\to\infty} \frac{n_{\bar{M}}|_{t\to\infty}}{n_{\bar{M}_p}|_{t\to\infty}} = k_{MLCK}|_{t\to\infty} \frac{1 - n_{\bar{M}_p}|_{t\to\infty}}{n_{\bar{M}_p}|_{t\to\infty}} \tag{4}$$

The remaining parameter values (k_3, k_4 and k_7) can be obtained by fitting the model via numerical simulation as shown in Section 3.2.

Contractile unit elongation

According to Figure 1, each CU consists of two actin filaments located above and below a myosin filament (lengths L_a and L_m), where the CU length L_{CU} is given by the distance between two consecutive dense bodies located between the myosin filament. Regarding to the myosoin cross-bridges, these are equally spaced by a distance δ. Changes in the CU length

are determined by the contribution of two components: u_{fs} associated to the relative filament sliding which can be generated by the myosin power-stroke or external deformation, and u_e that takes into account the elastic elongation of the cross-bridges attached to the actin filaments (i.e., u_e only exists when there are crossed bridges attached to actin filaments). Therefore, CU elongation is defined by:

$$\lambda = \frac{l_{CU}}{L_{CU}} = \frac{L_{CU} + u_{fs} + u_e}{L_{CU}} = 1 + \bar{u}_{fs} + \bar{u}_e \tag{5}$$

where u_{fs} and u_e are taken as positive values for relaxation, L_{CU} is the reference length of the CU, l_{CU} correspond to the current length of the CU, $\bar{u}_{fs} = u_{fs}/L_{CU}$ and $\bar{u}_e = u_e/L_{CU}$. In addition, L_o is the overlap between the actin and myosin filaments which can be defined as a function of u_{fs} (it is assumed that u_e does not contribute to the overlap). Another established assumption is that $L_a > L_m$, which defines three situations with respect to the overlap of filaments (Figure 1). In the first case, from the initial overlapped length $L_o(u_{fs} = 0) = x_o$ where there are not sliding effects ($u_{fs} = 0$), $L_o(u_{fs})$ increases as the actin and myosin filaments slide relative to each other. Continuing this process, the second situation occurs when the entire length of the myosin filament has been overlapped by actin filament, reaching (under a specific u_{fs}^{opt} value) its optimal value L_o^{opt} equals to the myosin filament length (L_m). In this situation, the optimal overlapped length (L_o^{opt}) remains constant until the analysed actin filament starts to move away from the myosin filament and the next CU appears, taking place the third situation, and this process is repeated again. This behavior can be modelled by a parabolic function, defining an optimal relative sliding filament value u_{fs}^{opt} as:

$$L_o = u_{fs} - \frac{u_{fs}^2}{2u_{fs}^{\text{opt}}} + x_o \tag{6}$$

It is also assumed that the active stress is directly proportional to the overlap length (L_o) for which the reference (active stress P_0)) and the optimum (active stress P_{opt}) states are

considered. From this consideration, it is possible to have an expression for the initial overlap (in the reference state):

$$\bar{x}_o = \frac{P_0}{2(P_{\text{opt}} + P_0)} \bar{u}_{fs}^{\text{opt}} \tag{7}$$

where $\bar{x}_o = x_o/L_{CU}$ and $\bar{u}_{fs}^{\text{opt}} = u_{fs}^{\text{opt}}/L_{CU}$.

It is worth mentioning that the filament sliding rate ($\dot{\bar{u}}_{fs}$) is defined in this model in terms of other variables that have not yet introduced and are described below in the present section.

Active stress

From the definition of the cross-bridge elastic elongation u_e (Murtada et al., 2012), an expression of the active stress P_a can be established as:

$$P_a = \mu_a \bar{L}_o (n_{AM_p} + n_{AM})(\lambda - \bar{u}_{fs} - 1) \tag{8}$$

where u_e is written in terms of the stretch λ and the relative filament sliding $\bar{u}_{fs}$ according to Expression 5. Adittionally, two terms are defined: $\bar{L}_o = L_o/L_{CU}$ which is a function of the relative filament sliding ($\bar{u}_{fs}$) (see Expression 6), and μ_a which is associated with the cross-bridge stiffness. It should be noted that Expression 8 involves the chemical part of the model along with the mechanical part, where $(n_{AM_P} + n_{AM})$ is obtained from a chemical analysis of the phosphorylation/ dephosphorylation process according to Expression 1, while the filament sliding process is described by the rest of the factors appearing in Expression 8.

Displacement rate

In order to get an approach to describe the contractile mechanism, an evolution law of the relative filament sliding (u_{fs}) is proposed according to the Hill muscle model for which a hyperbolic force-velocity relationship is written as:

$$\dot{\bar{u}}_{fs} = \beta_1 \frac{P_a - P_c}{P_a + \alpha} - \beta_2 \frac{P_a - P_c}{P_a - P_{LBC}} \tag{9}$$

where α, β_1 and β_2 correspond to fitting parameters, while P_{LBC} is refered to the maximum stress that can be supported by a CU. The second term in the right hand side of Expression 9 has been added to quantify the effect of muscle relaxation. On the other hand, the P_c, that operates as driving stress, depends on muscle state (relaxation or contraction):

$$P_c = \begin{cases} P_c^{cntr} = \kappa_{AMp} \bar{L}_o(\bar{u}_{fs}) n_{AMp} & \text{if } P_a < P_c^{cntr} \\ P_c^{relax} = \kappa_{AMp} \bar{L}_o(\bar{u}_{fs}) n_{AMp} + \kappa_{AM} \bar{L}_o(\bar{u}_{fs}) n_{AM} & \text{if } P_a > P_c^{relax} \\ P_a & \text{if } P_c^{cntr} \leq P_a \leq P_c^{relax} \end{cases} \tag{10}$$

In contraction, P_c^{cntr} depends on the overlapped length between myosin and actin $\bar{L}_o$, along with the fraction of those cross-bridges in a phosporylated state that are attached to the actin filament n_{AMp}. Muscle contraction state is possible if $P_a < P_c^{cntr}$. Meanwhile, the relaxation state P_c^{relax} depends on variables aforementioned to define P_c^{cntr} along with the attached and dephosporylated cross-bridge fraction (referred as latch state) (n_{AM}), related to a drop in the intracellular calcium level. Muscle relaxation state is possible if $P_a > P_c^{relax}$. When $P_c^{cntr} \leq P_a \leq P_c^{relax}$, P_c is defined by the active stress P_a, according to Expression 8.
